# Physical Couple and Family Violence Among Clients Seeking Therapy: Identifiers and Predictors

**DOI:** 10.3389/fpsyg.2019.02847

**Published:** 2019-12-17

**Authors:** Rune Zahl-Olsen, Nicolay Gausel, Agnes Zahl-Olsen, Thomas Bjerregaard Bertelsen, Aashild Tellefsen Haaland, Terje Tilden

**Affiliations:** ^1^Sørlandet Hospital, Kristiansand, Norway; ^2^Faculty of Health and Welfare, Østfold University College, Fredrikstad, Norway; ^3^Kristiansand Municipality, Kristiansand, Norway; ^4^Modum Bad Psychiatric Center, Vikersund, Norway

**Keywords:** partner violence, family violence, intimate partner violence (IPV), domestic violence (DV), physical violence, clinical sample, couple and family therapy

## Abstract

**Introduction:**

Couple violence (CV) affects many, and the consequences of those actions are grave, not only for the individual suffering at the hand of the perpetrator but also for the other persons in the family. Violence often happens among more than just the adults within one family. Even if CV has been thoroughly investigated in the general population very few studies have investigated this objective on a clinical sample, and none of these have included family violence.

**Aim:**

This article identifies and describes the group of clients that have issues of physical couple and family violence. It analyses a model that can help to discover physical violence and help therapists to assess what actions to take in therapy to prevent further physical violence.

**Methodology:**

Descriptive analysis, *t*-tests, and structural equation modeling (SEM) are used on a sample of clients receiving couple and family therapy (CFT) in Norway (*N* = 830). Family violence is modeled by the partner’s expectations toward each other, levels of anger, sexual satisfaction, and self-control.

**Results:**

One-in-five clients experienced physical CV in their current relationship and one-in-four experienced physical family violence. The group of clients who experienced CV differed from those without such experiences in having lower income, more prior experience with psychotherapy, more experience with alcohol abuse in childhood, and far more physical family violence in their current family. Our model predicting physical couple and family violence explained as much as 53% of family violence and had three positive, significant predictors (expectation, anger, and sexual satisfaction) and one, significant negative predictor (self-control). Somewhat unexpected, sexual satisfaction was a positive, and not a negative, predictor of violence.

**Conclusion:**

Our study identified one-in-four clients in CFT experience physical CV. Unreasonable expectation from one partner toward the other, anger and sexual satisfaction were positive predictors of physical violence, while self-control was found to be a negative predictor of physical violence. Implications for therapeutic work and the prevention of physical violence are discussed.

## Introduction

Partner violence is one of the most hurtful and traumatic experiences a human can experience as it shatters interpersonal trust and sense of safety (Stacey et al., 1994). Disturbingly, the prevalence of partner violence in the population has proven to be very large. An international population survey of physical abuse including 48 countries found that as many as 69% of women state that they have been subjected to physical abuse by a partner at some time of their life, but the results varied widely by country (Krug et al., 2002). Although violent episodes are often thought of as a clear-cut case with a powerful perpetrator and a powerless victim, most violence is to some extent reciprocal (Gausel et al., 2018) and in fact, several studies have found that as many males as females are exposed to partner violence (Haaland et al., 2005; Jose and O’Leary, 2009; Capaldi et al., 2012; Thoresen and Hjemdal, 2014). Indeed, studies from the United States have found that up to 61% of clients seeking couple and family therapy (CFT) have experienced couple violence (CV) (Jouriles and O’leary, 1985; Cascardi et al., 1992; Vivian and Malone, 1997) and it is reasonable to believe that these numbers are relevant for other comparable countries as well, such as Norway (Ormhaug et al., 2012). However, current studies have solely focused on CV and did not investigate the family violence that most often co-occurs and includes children. Hence, in the present study, we wanted to investigate both the physical CV and the physical family violence within a CFT sample.

In intimate relationships, such as family relations and couple relationships, there are extensive opportunities of experiencing and committing moral failures. Some of them are minor (e.g., forgetting important appointments), but others are more severe and some even illegal (e.g., abuse and violence). Naturally, committing acts of violence within families and couple relationships represents a grave moral failure (Gausel et al., 2018) that seriously question your integrity as a moral person (Gausel and Leach, 2011). However, having been violent might lead the perpetrator to be concerned for their social-image as a moral person, and thus fear being condemned by others, especially if the use of violence is in risk being exposed to these others (Gausel and Leach, 2011). According to Gausel and Leach (2011) and Gausel et al. (2018) this should promote even more violence in order to hinder the victim from exposing it or telling others about it. However, being victim to violence is a traumatic experience as well (Stacey et al., 1994) as it seriously question who you are as a person, especially your worth as a fellow human (Loring, 1994). Due to this, it is often appraised as a result of deep disrespect and because of this, it often promotes reciprocal violence (Gausel et al., 2018). In support of this, Gausel et al. (2018) conducted a field experiment in Liberia, Africa, with survivors of civil-wars that had been associated with groups that had been both victims and perpetrators of grave violence. In this study they argued for, and found, that when these survivors were encouraged to reflect on their victim-episodes they were significantly more motivated in seeking revenge than if they were encouraged to reflect on their perpetrator episodes. Hence, we find this especially useful in our context of couple and family violence (i.e., domestic violence) as the involved parties are both perpetrators and victims of violence, a type of violence that is suggested to be the most prevalent type of domestic violence (Fusco and Fantuzzo, 2009; Stith et al., 2011).

Even though CV primarily occurs among adults, it affects and includes the children severely. In fact, a Norwegian national survey (Haaland et al., 2005) found that 30% of the children witnessed the CV that had occurred, while an American study (Fusco and Fantuzzo, 2009) found that as many as 95% of the children had been exposed to violence exercised within the family. Of these children, 75% had an active role in trying to influence the situation by contacting a neighbor, calling the police, or protecting the victims of violence with their own bodies. Being a witness to violence profoundly affect children (Appel and Holden, 1998; Slep and O’leary, 2005; Kimball, 2016; Øverlien and Holt, 2018). In line with this, a meta-analysis by Kitzmann et al. (2003) concludes that 63% of children exposed to violence develop internalizing (e.g., posttraumatic stress disorder) and externalizing (e.g., aggression) problems. Hence, this group of children could be expected to become clients in children and adolescence clinics. Furthermore, children who experience violence in their childhood have higher risks of using violence themselves (Raundalen, 2009) and being exposed to violence in their adult lives (Wolf and Foshee, 2003; Renner and Slack, 2006; Øverlien, 2012). Hence, there is a high risk that the problem of violence gets passed from one generation to the next.

Obviously, therapists cannot offer appropriate treatment for CV or family violence if it is not detected, and unfortunately research shows that clients often do not inform their therapist about violence when they seek help (Ehrensaft and Vivian, 1996; Middelborg and Samoilow, 2014). Hence, several authors have recommended the use of universal screening for CV. Nevertheless, most therapists do not adhere to these recommendations in their clinical practice (Schacht et al., 2009; Todahl and Walters, 2011). We have not found anyone suggesting universal screening for family violence although it occurs so frequently. The lack of such screening implies that violence is often not discovered and thereby not treated (Stith et al., 2011). This seems to be the case also when a child is the index patient. Reigstad et al. (2006) found that 60% of the children in their study had experienced physical violence in their family, but that was reported in the journaling system only in 0.4% of these cases. Thus, it was not discovered or not found to be important for the treatment. The lack of couple and family violence disclosure within the CFT field represents a professional challenge, in particular since CFT treatments are found to be effective treatments for CV (Stith et al., 2012).

To help clinicians detect cases where violence is part of the problem, even if it is not explicitly mentioned in the referral or by the clients, therapists need knowledge of theory, typical patterns of partner violence (see e.g., Walker, 1979; Bensimon and Ronel, 2012; Entilli and Cipolletta, 2017), and predictors of couple and family violence. Knowledge of predictors of couple and family violence can also help therapists understand the complexity of this type of violence and thereby provide more appropriate therapy. Moreover, developers of treatment for couple and family violence can use the predictors to tailor new and possibly more efficient treatments.

Even if predictor studies of couple and family violence are rare on a clinical CFT sample, predictor studies of CV have been conducted on other samples [see e.g., O’Leary and Woodin (2009) for a review]. Unlike many other health problems, few social and demographic characteristics define risk groups for intimate partner violence (Jewkes, 2002). Nevertheless, CV relates to other personal and relational factors. Low income and low level of education have been found to be associated with higher prevalence of CV (see e.g., Pan et al., 1994; Stith et al., 2011). Furthermore, it has been determined that physical CV is associated with alcohol and substance abuse, both at present time and in adults’ family of origin (FOO) (e.g., Coker et al., 2000; Fals-Stewart, 2003; Fals-Stewart et al., 2005; Stith et al., 2012). In addition, the use of violence in close relations is confirmed to be closely related to anger (Simpson et al., 2007).

A close relationship between physical and sexual abuse has been found in several studies (e.g., Simpson et al., 2007, 2008; Stith et al., 2012). However, we could not find any study that investigated the relationship between sexual satisfaction and CV. Issues of sexual satisfaction are common in CFT treatment. Since good sexual satisfaction is found to be associated with relationship satisfaction, love, commitment, and stability (Young et al., 1998; Sprecher, 2002; Butzer and Campbell, 2008), it is reasonable to believe that there is less CV when the sexual satisfaction is high. Most couples argue and fight over practical issues like household chores, and it is common that some of these issues occur repeatedly (Gottman and Silver, 2015). These authors argue that the underlying issues are often values or expectations: e.g., if the expectation of one partner about the other partner’s household chores is higher than what the other expects of herself, there is likelihood of conflict. This could lead to tiring arguments with high tension or even violent outcomes. However, different people have different conflict management styles that have been assessed in relation to CV (see e.g., Vivian and Malone, 1997; Simpson et al., 2007; Gottman and Silver, 2015), and the findings suggest some styles of conflict management to be far more associated with violence than others. The authors define these in different ways, but there is a consensus that self-control is viewed as a positive asset in high conflict.

As mentioned, the knowledge of couple and family violence within a clinical CFT sample is limited. There are even fewer studies of predictors of couple and family violence within this group of clients, and no studies where family violence has been included. In this study, we investigated some characteristics among CFT clients with defined issues of violence. Furthermore, we investigated a model that may be helpful in discovering couple and family violence, thus helping therapists to assess what actions to take in therapy to prevent further physical violence. To our knowledge, this is the first study to investigate CV on a clinical CFT sample outside the United States, and the first study to investigate predictors of both physical CV and physical family violence in a CFT sample.

The research questions are:

(1) What is the prevalence of physical couple and family violence in a clinical sample?
(2) What identifies clients who experience physical CV?
(3) What predicts physical couple and family violence?

## Materials and Methods

We have conducted a study of clients seeking CFT treatment by using quantitative data collected with the Systemic Therapy Inventory of Change (STIC) (Pinsof et al., 2009) feedback system. STIC measures several aspects of clients’ lives including physical couple and family violence together with several items that possibly can predict this violence.

### Sample

The initial sample for this study consisted of 830 clients above 18 years of age [mean age 40.3 years (SD = 8.5); age range 18–72 years; 51.8% were women, mean 2.3 children (SD = 1.1), see the table in the Supplementary Material for more details].^1^ Data collection started in March 2010 and was ended in April 2016 and the sample consists of data from all three levels in the stepped level of care within CFT in Norway. The first and second levels of care were represented by outpatient agencies. At the first level, no referral was needed. The third and final level of care was represented with an inpatient CFT agency, where a referral was needed. In all, 56% (462) outpatients and 54% (368) inpatients were included in the study. All participants completed the STIC (Pinsof et al., 2009) initial questionnaire in Norway during a pilot (Tilden et al., 2015) and an RCT study (Tilden et al., 2019).

### Ethics

Written informed consent for collecting the project data was obtained from each participant. This study was approved by the Modum Bad Ombudsman for Data Protection and the Regional Ethics Committee for Medical Research with human subjects (2017/96/REK sør-øst C). The primary study is registered at ClinicalTrials.gov. Since the data originate from regular clinical practice, no inclusion or exclusion criteria have been used except for the ones each site has for accepting clients for treatment.

### Measurements

#### Measures

Systemic Therapy Inventory of Change (Pinsof et al., 2009) is an assessment and feedback system in which clients fill out a questionnaire before each therapy session. Via electronic devices, the clients evaluate their response to treatment, including progression, and alliance to the therapist (Pinsof et al., 2009; Tilden et al., 2010). Client evaluations are processed into a report that is returned to the therapists who can use this information as the basis for understanding and hypotheses in the clinical assessment of the current client. The response options in STIC are on a five-level scale from worst to best option. Modules are added depending on the therapy mode and number of clients in the therapy system. The questions cover six subscales, individual problems and strengths (IPS), FOO, relationship with partner, family/household, children’s problems, and strength and relationship with child or children. On average, it takes 45 min to complete the STIC questions before the first treatment session. Before every subsequent session, the clients complete a short version of STIC that takes 7–8 min to fill out. Because this study is cross-sectional by making use of data before the first session only, the intersession data were not included. STIC has good internal reliability (Pinsof et al., 2009; Zinbarg et al., 2018; Zahl-Olsen et al., in review) with a Cronbach’s alpha as high as 0.94 for the different subscales.

The STIC consists of several subscales that further contain factors and items, and some of these address the topic for this study. The response options to those questions were, not at all/never, rarely, sometimes, often, and all of the time. The relationship with partner (RWP) scale has one item addressing physical violence between the members of the couple: “We get into shoving or hitting each other when we fight.” The family household (FH) scale has two items addressing physical violence exerted within the family: “Someone in my family is physically abusive to other family members” (item 1) and “There is someone in my family who pushes other family members around physically to get his or her way” (item 2). We combined these two items to one family violence item in our analysis. We hypothesized that both the CV item and the family violence item would load from the same latent variable Physical Violence. Further, the IPS scale, the RWP scale, and the FOO scale all have variables we, based on the presented theory and the literature review above, hypothesize are predictors for the underlying latent variable – Physical Violence. We also modeled that perceived anger toward the partner and level of expectation of household chores were predictors of Physical Violence. We hypothesized two negative predictors of Physical Violence: Self-control of thoughts, impulses, and rage as the first one, and sexual satisfaction within the relationship as the second. See Table 1 for a full list of items included in the variables.^2^

**TABLE 1:** List of items used to model physical violence.

	**Variable**	**Item name**	**Items in the STIC questionnaire**
**Physical violence**	Couple violence		We get into shoving or hitting each other when we fight
	Family violence		Someone in my family is physically abusive to other family members
			There is someone in my family who pushes other family members around physically to get his or her way
**Predictors**	Anger		I am filled with anger toward my partner
	Expectation	Expect 1	My partner often acts like he or she can’t stand me
		Expect 2	My partner often complains that I don’t do my share of work around the house
		Expect 3	I am expected to do too much.
	Sexual satisfaction	Sexsat 1	I am sexually frustrated in this relationship
		Sexsat 2	I am sexually satisfied with my partner
	Self-control	Selfcont 1	Had urges or impulses that you could not control
		Selfcont 2	Had fits of rage you could not control

### Statistical Analysis

We used IBM SPSS Statistics 25 for descriptive, bivariate, and multivariate analysis, and Amos 25 for structural equation modeling (SEM) analysis. Descriptive, correlation, *t*-tests, and crosstab analysis were performed as instructed by Field (2018) to describe the sample, calculate the statistics, and to compare the group of clients who indicated CV with the clients who did not. Further, the statistical analyses included two multivariate general linear models, which were performed as described by Field (2018), where existence of CV was set as the fixed factor.

In this study, we used SEM to predict Physical Violence. This method has several advantages compared to more conventional statistical techniques (Kline, 2016). For example, in multiple regression, it is an assumption that all predictors are measured without errors. This is routinely violated in practice, but when using SEM, we can make explicit representations of measurement errors. SEM also makes it possible to model the correlations between the variables and include that as part of the analysis. Finally, SEM involves significance testing of whole statistical models and not just individual effects (Kline, 2016). Based on our expectations, we modeled the latent variable Physical Violence, which is expressed as physical violence between the couple and physical violence between others in the family. Further, we hypothesized that predictors in the model were the partner’s expectations toward each other, levels of anger, sexual satisfaction, and self-control, as described below.

## Results

### Descriptive Analyses

Our first approach to the research question addressing the prevalence of couple and family violence was to analyze the clients’ responses to the item “We get into shoving or hitting each other when we fight.” As many as 20.4% (*n* = 169) confirmed that this described their relationship from “rarely to all of the time.” This group is from now on called the CV group as discriminant from the rest of the group labeled no couple violence (NCV) group. Within the CV group 84% (142) reported rarely, 13% (22) sometimes, 3% (5) often, and 0% (0) all the time. Our next approach to the first research question was to analyze the clients’ responses to family violence. As many as 24.9% (207) responded from “rarely to all of the time.”

Our second research question addressing the identification of clients who experience physical CV was assessed by comparing the CV and the NCV groups. We found that the CV group had significantly more prior experience with therapy [χ^2^(3) = 8.165, *p* = 0.043] and had lower income [χ^2^(2) = 13.612, *p* = 0.001] compared to the NCV group. However, the two groups did not differ on measures of age and education. An important difference between the two groups was the presence of family violence. In the CV group, as many as 49.7% reported that family violence was present. This was significantly higher [χ^2^(1) = 84,324, *p* < 0.001] than in the NCV group reporting 18.0%. The CV group was significantly more distressed than the NCV group, on all the measures included in the model. Using Roy’s Largest Root, there was a significant effect of experiencing CV on couple and family violence, Θ = 6.34, *F*(2,691) = 2191.40, *p* < 0.001. For the violence items higher values are better and for family violence the sample of clients in the CV sample had a mean of 4.46 (SD = 0.61) and the NCV group 4.77 (SD = 0.55). For CV the sample of clients in the CV sample had a mean of 3.81 (SD = 0.46) and the NCV group 5.00 (SD = 0.00). Further, using Roy’s Largest Root, there was a significant effect of experiencing CV on the predictors in the model, Θ = 0.19, *F*(4,762) = 35.33, *p* < 0.001. For Anger and Expectation higher values are better and the mean values on Anger were 3.20 (SD = 0.89) for the CV sample and 3.82 (SD = 0.97) for the NCV sample. The mean values on Expectation were 2.89 (SD = 0.81) for the CV sample and 3.56 (SD = 0.77) for the NCV sample. For Sexual satisfaction and Self-control lower values are better and the mean values for Sexual satisfaction were 2.87 (SD = 0.99) for the CV sample and 2.66 (SD = 1.08) for the NCV sample on. The mean values for the CV sample were 1.96 (SD = 0.83) and 1.63 (SD = 0.71) for the NCV sample on Self-control. See the table in the Supplementary Material for further information.

### Model Analysis: Structural Equation Modeling

**FIGURE 1:**
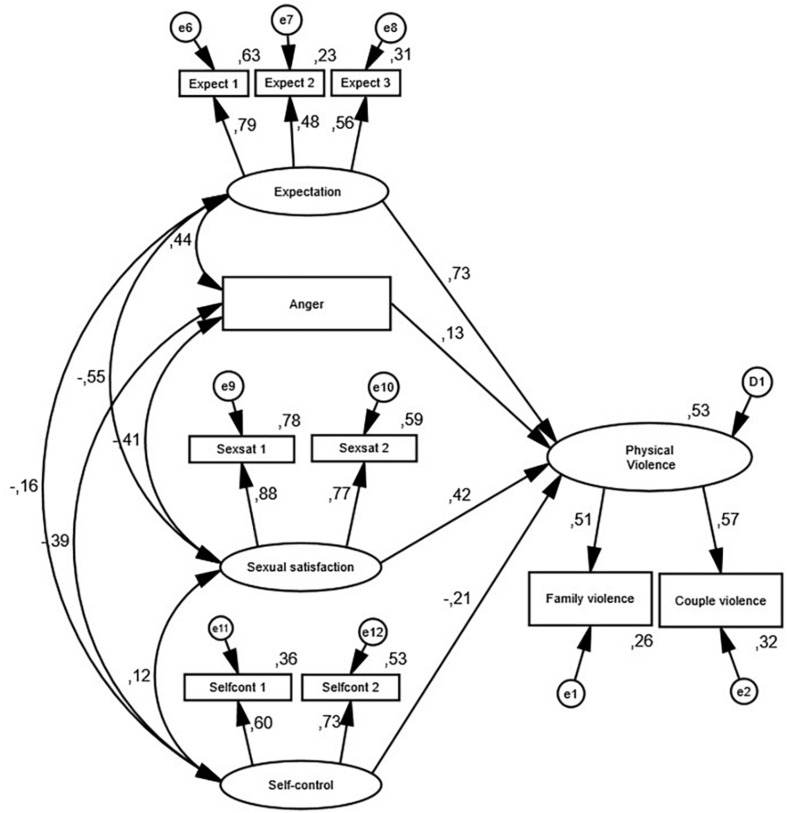
Predictive model of Physical Violence. All covariances and factor loadings were statistically significant (*p* < 0.05).

Our third research question addressed possible predictors of couple and family violence in the total sample. The model was constructed with the variables as previously described^3^, with four predictors and one latent variable, Physical Violence, as the outcome variable. The model as displayed in Figure 1 explained 53% of the change in Physical Violence and demonstrated very good fit for this sample. The chi-square was: χ^2^(27) = 78.672, *p* = 0.000, which indicates that the model fits the data well. However, given the complexity of the model, chi-square is an inadequate test of model fit (Kline, 2016). A better way to test how well our hypothesized model fit the data is provided by a χ^2^/df ratio below 3 (2.914) and several chi-square-based fit indices above 0.900 [incremental fit index (IFI) = 0.967, comparative fit index (CFI) = 0.967]. In addition, good model fit was shown by our observation of a residual index, where lower is better [root-mean-square error of approximation (RMSEA) = 0.048 with the confidence intervals, 0.036–0.061; see Kline, 2016]. The model was stable also when controlling for demographics.^4^ The standardized solution is shown in Figure 1^5^, and the scale inter-correlations and descriptive statistics are presented in Table 2.

**TABLE 2:** Scale inter-correlations and descriptive statistics.

	**1**	**2**	**3**	**4**	**5**	**6**
1. Expectation	–					
2. Anger	0.31^∗∗^	–				
3. Sexual satisfaction	–0.36^∗∗^	–0.37^∗∗^	–			
4. Self-control	–0.11^∗∗^	–0.31^∗∗^	0.09^∗∗^	–		
5. Family violence	0.21^∗∗^	0.12^∗∗^	0.00	–0.11^∗∗^	–	
6. Couple violence	0.30^∗∗^	0.25^∗∗^	–0.05	–0.17^∗∗^	0.28^∗∗^	–
Mean	3.41	3.68	2.71	1.69	4.70	4.74
SD	0.82	0.99	1.06	0.74	0.57	0.54
α	0.65	–	0.81	0.61	0.85	–

*^∗∗^*p* < 0.01.*

The two strongest predictors of Physical Violence were Expectation (β = 0.73, *p* < 0.001) and contrary to our expectation, Sexual satisfaction (β = 0.42, *p* < 0.001). Anger was also a positive predictor (β = 0.13, *p* < 0.001). Self-control was a statistically significant negative predictor of Physical Violence (β = −0.21, *p* = 0.004). The correlation analysis showed that all the predictors were statistically significant related to each other (all *p*s < 0.05). The medium levels of correlation and the variation of the correlation coefficients indicate that the included variables measure different aspects of the client’s life.

## Discussion

We found that 20.4% of the clients reported that physical violence occurs in their relationship. Furthermore, as many as 49.7% of the clients reporting physical CV also reported physical family violence. The clients with physical CV had lower income and more prior experience with therapy. The strongest predictor of Physical Violence were Expectation, while Self-control was a negative predictor.

### Prevalence of Physical Couple Violence in a Clinical Sample

The prevalence of CV in the clinical CFT sample in the current study is 20.4% which is more than four times as high as the prevalence of CV in the general population in Norway (Haaland et al., 2005). More precisely, this study identifies physical violence and not a more generally defined violence. Furthermore, in this study, we have specified the questions with regard to the current relationship. This is contrary to most population studies, which ask if people have experienced CV in *any* intimate relationship until now. US studies of couples seeking therapy have found frequencies of CV up to 61% (Jouriles and O’leary, 1985; Cascardi et al., 1992; O’Leary et al., 1992; Vivian and Malone, 1997; Simpson et al., 2007; Stith et al., 2011). However, in these studies, they have used a more general definition of CV that includes psychological, physical, and sexual violence without differentiating between these. Hence, more research is needed where the types of violence are accounted for.

### Couple Violence and Family Violence Occurs Simultaneously

Our descriptive analyses found that there is a significantly higher distribution of family violence in the CV group than in the NCV group. This co-occurrence of violence in the couple and violence in the family is in line with the findings of Slep and O’leary (2005) and Appel and Holden (1998). However, the high prevalence of both couple and family violence among clients seeking CFT identified in our study indicates that every therapist meets clients with these issues frequently, even if it is not addressed. Further efforts should therefore be made in CFT to discover and end couple and family violence than is the case currently. Since some research indicates that clients are more likely to reveal family violence when responding to a questionnaire compared to when questioned face to face by a therapist, e.g. (Ehrensaft and Vivian, 1996; Andersen and Svensson, 2013; Zahl-Olsen and Oanes, 2017), we suggest implementing systematic screening for both couple and family violence, for instance as part of a feedback system.

Family violence co-occurs with CV for several reasons. Edleson (1999) found that children were involved in the violence among the adults, while Raundalen (2004) found that children tried to intervene to stop the violence. Further, parents who conduct physical violence against their partner also do so toward the children in the family (Appel and Holden, 1998; Slep and O’leary, 2005). It is found that children exposed to severe anger and aggression in their domestic environment increase their risk of becoming more aggressive (Raundalen, 2009). In addition, children exposed to violence in their home have higher risks of experiencing violence with others later in life compared to those who did not encounter such violence in their childhood home (see, e.g., Wolf and Foshee, 2003; Øverlien, 2012). Indeed, children exposed to violence in their childhood also have a greater risk of being exposed to adult violence (Renner and Slack, 2006) later in life. Thus, there is a high risk that violence is transferred from one generation to the next. Finally, these findings support the theoretical argumentation of Gausel and Leach (2011). In their theoretical framework, children who are exposed to violence in their home might appraise this violation as a sign of threat to their worth, and by such, a sign of disrespect. However, it can also be appraised as a global defect in their family, i.e., we are violent people, which again might provoke anger. Combined, these two ways to appraise violence might encourage further violence to end the ongoing disrespect, a last effort to protect their social image as someone who is worthy of respect (Gausel, 2013; Gausel et al., 2018).

### Differences Between the Samples With and Without Couple Violence

In our study, the prevalence of couple and family violence is higher among those with low income. This is a finding similar to other studies, see e.g., Weede (1981), Gelles (1997), and Rennison and Planty (2003). Further, it is an interesting finding that the CV group has received more prior treatment than the NCV group. However, this might indicate that the CV group consists of families with more severe issues than the NCV group and therefore needs more treatment. If so, it is an empirical question whether the treatment they received was helpful if the violence was addressed as a topic in therapy, or whether – as previously mentioned – the therapeutic interventions so far did not reveal the ongoing violence. There were significant differences between the CV sample and the NCV sample on the levels of distress on the predictor variables. However, the differences were small and both groups indicated distress at all four predictors. Therefore, we continued to investigate the individual differences on the total sample.

### Expectation and Anger

Our model demonstrated that the experiences of unreasonable expectation from one partner toward the other in relation to household chores were the strongest predictor of Physical Violence. In other words, the more experiences of unreasonable expectation from the partner, the more physical violence in the relationship. This is in agreement with research finding that marital discord serves as a strong predictor of both mild and severe husband-to-wife physical aggression (Pan et al., 1994; Stith et al., 2008; Capaldi et al., 2012). However, since males and females are found to be equally exposed to CV (Haaland et al., 2005; Jose and O’Leary, 2009; Stith et al., 2011; Thoresen and Hjemdal, 2014) our study did not differentiate between husband-to-wife and wife-to-husband aggression.

When viewing this strong prediction from Expectation to Physical Violence from the theoretical perspective of Gausel and Leach (2011) we understand why this link is so strong. When someone experience that the other person in their relationship is viewing them as someone who does not manage to live up to what is expected, they might feel inferior, and thus wanting to act, either to withdraw, which is hard in a close relationship, or by using verbal or physical force.

We found that expectation and anger were significantly correlated. It makes sense that when a partner experiences herself or himself to be criticized and treated unfairly, he or she experiences anger (Isdal, 2000). The other way around also makes sense. Gottman and Silver (2015) describe how a person with anger and arousal interprets even neutral words as negative. This is also in line with the conceptual theory of Gausel and Leach (2011) that being criticized can lead to a feeling of inferiority and rejection which again is associated to anger. However, anger in our model is the third strongest predictor of Physical Violence. Apart from cases where dominating the other partner is the issue it is hard to think of CV occurring without anger, but it is important to point out that experiencing the feeling of anger does not say it has to lead to physical CV (Leone et al., 2007). Leone et al. (2007) argue that violence is usually about lack of affective regulation, communication difficulties, and the lack of skills for problem-solving. Thus, if a person has not learned to be conscious of and learned how to handle basic feelings such as anger, this can, in turn, lead to unwanted behavior like physical violence.

### Sexual Satisfaction

Among our two expected negative predictors for Physical Violence, better Self-control, and higher Sexual satisfaction, only the first was confirmed. Moreover, our SEM analyses indicated that Sexual satisfaction in the relationship predicted Physical Violence. This is contrary to our expectations and contrary to studies of sexual satisfaction elsewhere in the field of CFT where sexual satisfaction is found to be associated with good and healthy relationships (see e.g., Sprecher, 2002; Litzinger and Gordon, 2005; Yeh et al., 2006; Butzer and Campbell, 2008). Besides, Litzinger and Gordon (2005) found that sexual satisfaction partially compensates for the negative effects of poor communication. However, one possible way to understand our finding that higher sexual satisfaction predicts CV is that couples who emotionally live distant from each other have very little contact and do not argue and fight. In consequence, they do not have much sexual intimacy either. This group might level out those who occasionally fight and have a satisfactory sexual life. This inference is supported by the fact that 84% (142) of those indicating physical CV answered that the physical violence occurred seldom. Furthermore, we found a normal distribution of sexual satisfaction within the group who indicated that they seldom experienced CV. Walker’s (1979) cycle of violence identifies a *honeymoon phase* that follows a phase of acute battering. In this honeymoon phase, identified as calm and loving, satisfying sex can be present. This could explain why sexual satisfaction came out as a predictor of physical violence in this study. However, we have not been able to find research on the direct connection between sexual satisfaction and violence in relationships. Hence, we suggest further research on the relationship between sexual satisfaction and CV. Finally, the conceptual model of shame (Gausel and Leach, 2011; Gausel, 2013; Gausel et al., 2018) explains that if the moral failure is appraised as a self-defect that is mendable the person will try to repair what is broken. Sex could be a way to strengthen and restore one’s self-image as someone to love and respect, and even more to build stronger social bonds to the partner and thus prevent condemnation.

### Self-Control

As mentioned, self-control was found to be a negative predictor of Physical Violence. Thus, the more self-control the clients has the lesser physical violence they experience. Furthermore, this seems to be a variable that is not closely related to the other variables in our model. In other words, this may be a variable that is stable even when the others vary. This seems especially true in relation to sexual satisfaction and expectation. Thus, from a clinical point of view, strengthening the clients’ self-control could contribute to bringing stability into the relationship and decreasing physical violence.

### Strengths and Limitations

It is a strength of our study that we have data from all three different levels of CFT treatment in Norway, including samples from low threshold outpatient clinics without the need of a referral, an outpatient clinic where referral is needed, to an inpatient clinic where referral is needed. However, that the sample, thereby, is heterogeneous could also be considered a limitation because we have not analyzed the differences between the clinics. A second strength is the size of the data set and furthermore that approximately half of the data stems from an RCT with strict control of data collection. A third strength is that the type of violence is specified as physical violence in the current relationship. However, it is a limitation that the clients did not report who exercised the CV: whether it is themselves as perpetrators, whether they are exposed to it by their partner, or whether it is both. A second limitation is that we could not differentiate between severe physical violence and minor physical violence. However, one could argue that by dividing the sample into three groups rather than two we could gain more information: 1 = no violence, 2 = seldom violence, 3 = often or more. However, we had two reasons for not doing so. First, even if violence seldom occurs, we do not know how severe it is, and a few very severe incidents might be as powerful as many less severe. Second, if violence only rarely occurs it can still be an important part of their family life. A third limitation is the narrow focus on anger as the only emotion identified as predictor of family violence in our model. Undoubtedly, there are other emotions, such as a feeling of rejection (Leary et al., 2006; Gausel, 2013) or of hate (Staub, 2005), that predict violence and anti-social behavior. We acknowledge that we are not able to identify whether one or both parties are conducting violence. However, recent research on violent conflicts suggest that in most violent conflicts both parties exercise violence but prefer to construe themselves as victims instead of perpetrators (e.g., Mazziotta et al., 2014; Gausel et al., 2018). By such, it is not unthinkable that if we had been able to explore who exercised violence in our study parties would tend to see themselves as victims and the other as the perpetrator – therefore skewing the reports of violence toward the other instead of reporting their own perpetration (for a discussion, see Gausel et al., 2018). Some might question if the sample of clients who experienced physical violence is representative for this group, since 84% of them indicate that the physical violence is experienced rarely and 13% sometimes. We share this concern and want to make clear that this is how the clients have reported physical violence in their current relationship, and that under-reporting is possible. However, this sample might not be representative for clients, e.g., seeking therapy specialized at domestic violence or coming to women’s shelters.

## Conclusion and Clinical Implications

This study has found that the prevalence of couple and family violence in a clinical sample is high, indicating that many CFT therapists encounter this topic in therapy. Because this topic may remain undisclosed during treatment, we assume that a higher portion of CFT therapists may relate to this topic indirectly because when this remains a secret, it may impact a great deal on the presented topics in therapy. The notion that men cannot be victims of physical and psychological violence by their partners (see e.g., Entilli and Cipolletta, 2017) may impose a considerable barrier for therapists to interpret such signs. Since the immediate and consequential damages of couple and family violence are grave for the adult, their children, and their future generations, therapists need tools to discover these issues. Knowledge of the predictors in this study can possibly help to uncover physical abuse in couple relationships and families. Therapists who have little knowledge of physical violence and predictors of such violence risk to miss the opportunity to assess signs of an abusive relationship, or to seek further assistance from child protection services and police. Knowledge of predictors of physical violence can also bring the confidence in the CFT therapists to dare to ask more specific and handle the case in a proper way. CFT therapists are in contact with more couples and families where violence occur, than the police (see e.g., Reigstad et al., 2006; SSB, 2016) and are enforced to notify when this is uncovered (Barnevernloven, 1993). In Norway, this law also enforce teachers, social workers, and therapists to be aware of circumstances that are harmful (Driscoll, 2018). This challenges CFT therapists, especially those inspired by social constructionist theories which not want to enter areas in people’s lives that the clients do not directly address (Anderson, 2001). When it comes to violence we cannot allow therapists to be more influenced by their own perspectives than the data about the situation (Jensen, 2008). It is pivotal to enhance the knowledge of predictors of physical violence that could be noticed by CFT therapists and allow a more effective screening.

In addition, as shown in our model, we have addressed certain predictors who can help to discover physical violence in ordinary CFT practice. Furthermore, our findings could assist therapists in assessing what actions to take in therapy to prevent further physical violence. For example, by focusing the therapy on expectations toward each other’s participation in household duties and what lays behind those expectations. It is possible that, through therapy, focusing on attaining mutual understanding of the expectation toward each other this tension will go down and thus prevent physical violence. Further, as the conceptual theory of Gausel and Leach (2011) and Gausel et al. (2018) explains, the feeling of rejection, through the experiences of loss of social image (others’ view of self), and the feeling of inferiority can lead to violent acts. Hence, if the focus in therapy is on understanding and acceptation of the partners’ view of self, this will probably impact on reduced uncertainty about the partner’s view of self. Since most partners want to stay in the relationship even if violence is exerted (Stith et al., 2011) it is reasonable to think that their view of their partners is mostly positive. Reduced uncertainty implies fewer chances of viewing self-defects as global, instead increasing the chance that failures are specific and thus can be more easily dealt with. In return, as Gausel and Leach (2011) argue, this should lead to acts of reconciliation and amendments.

These findings address clinical implications on how to treat couples and families where abuse is a topic. However, it is outside the scope of this paper to give a thorough clinical guide on how to do so. There is a rich literature that the reader is encouraged to search for on this topic.

### Suggestions for Further Research

We suggest further research on the prevalence and predictors of different types of couple and family violence (verbally, sexually, and physically) in clinical samples. Based on our unexpected finding of the relationship between sexual satisfaction and physical violence we suggest investigating this relationship in further research. Our study has also found that alcohol abuse was a less important predictor of physical violence than the literature suggests. Thus, we encourage further research on aspects of expectation, anger, and self-control as predictors for physical couple and family violence.

## Data Availability Statement

The raw data supporting the conclusions of this article will be made available by the authors, without undue reservation, to any qualified researcher.

## Ethics Statement

Written informed consent for collecting the project data was obtained from each participant. This study was approved by the Modum Bad Ombudsman for Data Protection and the Regional Ethics Committee for Medical Research with human subjects (2017/96/REK sør-øst C). The primary study is registered at ClinicalTrials.gov. Since the data originate from regular clinical practice, no inclusion or exclusion criteria have been used except for the ones each site has for accepting clients for treatment.

## Author Contributions

RZ-O has been responsible for the design, analysis, drafting, and revising of the text. TT, AH, and RZ-O have been responsible for acquiring the data. NG and TB have been responsible for the analysis together with the main author. AZ-O has been responsible for preliminary analysis, searching for manuscripts and drafting the manuscript. TT, AH, and NG have been responsible for supervising the results and intellectual content.

## Conflict of Interest

The authors declare that the research was conducted in the absence of any commercial or financial relationships that could be construed as a potential conflict of interest.

## Footnotes

1 813 defined themselves as heterosexual, 8 as bisexual, and 2 as homosexual/Lesbian.

2 Since prior studies (see e.g., Coker et al., 2000; Fals-Stewart, 2003; Stith et al., 2012) indicated that alcohol abuse might be of importance and STIC provided measures of alcohol abuse at three different levels: for themselves, for their partner, and in their FOO, we wanted to assess if these were significant contributors to the model also in our clinical sample.

3 Results from the CFA on the model was χ^2^(15) = 64.211, *p* < 0.001. χ^2^/df ratio = 4.281, IFI = 0.964, CFI = 0.964, RMSEA = 0.063. The correlations were: 0.44 between Expectation and Anger, −0.55 between Expectation and Sexual satisfaction, −0.15 between Expectation and Self-control, −0.41 between Anger and Sexual satisfaction, −0.39 between Anger and Self-control, and 0.12 between Sexual satisfaction and Self-control. The factor loadings from Expectation was 0.80 to Expect 1, 0.47 to Expect 2, and 0.56 to Expect 3. The factor loadings from Sexual satisfaction was 0.88 to Sexsat 1 and 0.77 to Sexsat 2. The factor loadings from Self-control was 0.58 to Selfcont 1 and 0.75 to Selfcont 2.

4 None of the three alcohol abuse items improved the model.

5 Results from the analyses of the same model using the CV sample (*N* = 165) were *R*^2^ = 0.12, χ^2^(27) = 37.476, *p* = 0.087. χ^2^/df ratio = 1.388, IFI = 0.969, CFI = 0.966, RMSEA = 0.048. Analyzing the model on the NCV sample (*N* = 604) requires removal of the latent variable Physical Violence since CV is zero for all participants in this sample. Thus, the outcome variable is the manifest variable Family Violence. The results of this model were *R*^2^ = 0.04, χ^2^(19) = 63.127, *p* < 0.001. χ^2^/df ratio = 3.322, IFI = 0.956, CFI = 0.955, RMSEA = 0.062.
